# Effects of 12-week aquatic HIIT on blood pressure lipid profile and BaPWV in postmenopausal women with different ACE genotypes

**DOI:** 10.1038/s41598-026-36835-1

**Published:** 2026-01-28

**Authors:** Wen-sheng Zhou, Yu-hong Li, Na Xu, Bao-fang Liu, Zi-hao Li, Shuai-jie Gu, Rong-yao Zhang, Xuan-Xu, Cheng Yan, Qi-yu Wang, Lei Sun, Meng Gu, Tian-qi Zhu, Lei Tian

**Affiliations:** Department of Physical Education, Jiangsu Second Normal University, Nanjing, China

**Keywords:** Older women, Renin-angiotensin-aldosterone system, Metabolic syndrome, Atherosclerosis, Exercise genomics, Diseases, Genetics, Physiology

## Abstract

The angiotensin-converting enzyme (ACE) insertion/deletion (I/D) polymorphism influences renin-angiotensin-aldosterone system activity and may modulate cardiovascular adaptations to exercise. Yet, evidence regarding genotype-dependent responses to aquatic high-intensity interval training (HIIT) in postmenopausal women is limited. We aimed to compare the effects of 12-week aquatic HIIT on blood pressure, lipid profile, and arterial stiffness between postmenopausal women with the ACE II genotype and those carrying at least one D allele (ID/DD genotype). Sixty postmenopausal women aged 45–75 years were recruited, with ten participants voluntarily withdrawing from the study and three lost to follow-up. A total of 47 participants completed the intervention (21.7% attrition). Participants were stratified into ACE II (n = 25, 59.0 ± 5.52 years) and ID/DD (n = 22, 57.4 ± 7.52 years) genotype groups. Participants performed a 12-week aquatic HIIT program, with three 40-minute sessions per week. Each session consisted of a 6-minute warm-up, 30 min of main training (involving strength and jumping exercises), and a 4-minute cool-down. key cardiovascular outcomes were measured at pre- and post-intervention. Following a 12-week aquatic HIIT program, no significant differences were observed in post-intervention systolic blood pressure (SBP), diastolic blood pressure (DBP), or mean arterial pressure (MAP) between the II and ID/DD groups (all p > 0.05). While no significant between-group differences were found in brachial-ankle pulse wave velocity (baPWV) on either side (p = 0.058, 0.086), a greater magnitude of change in baPWV values was observed in the ID/DD group. Within-group analyses revealed that the II group exhibited significant reductions in SBP, DBP, MAP, and baPWV(right) (p = 0.023, 0.041, 0.020, 0.019), whereas the ID/DD group showed significant increases in baPWV (right/left, p = 0.013, 0.002). Post-intervention TG levels were significantly lower in the ID/DD group compared to the II group (p = 0.000), with a non-significant trend toward higher HDL-C levels (p = 0.053). Both groups demonstrated significant improvements in lipid profiles, characterized by increased HDL-C and decreased LDL-C (p < 0.05). The aquatic HIIT program significantly improved blood lipids in postmenopausal women, yet no significant ACE genotype-specific effects were observed on blood pressure or arterial stiffness. While the II group exhibited favorable reductions in blood pressure, the ID/DD group showed increased arterial stiffness, suggesting potential vascular risks and underscoring the need for monitoring during exercise.

*Trial registration*: ChiCTR2400087544 (July 30, 2024).

## Introduction

The decline in estrogen levels following menopause severely impairs the regenerative capacity of vascular endothelial cells, leading to endothelial dysfunction, reduced arterial compliance, and concomitant increases in blood pressure and dyslipidemia^1,2^. These changes are characterized by elevated levels of systolic blood pressure (SBP), diastolic blood pressure (DBP), mean arterial pressure (MAP), low-density lipoprotein cholesterol (LDL-C), and triglycerides (TG), along with reduced high-density lipoprotein cholesterol (HDL-C). Additionally, brachial-ankle pulse wave velocity (baPWV), a marker of arterial stiffness, may be elevated^3^. Collectively, these alterations significantly increase the risk of hypertension, arterial stiffness, and lipid metabolism disorders^4^. Postmenopausal women face a twofold higher risk of cardiovascular disease compared to premenopausal women, and the risk of stroke doubles within the first decade after menopause^5,6^. Consequently, interventions targeting cardiovascular risk in postmenopausal women have become pivotal in mitigating the global burden of cardiovascular disease^7,8^.

Hormone replacement therapy has been used to alleviate various health issues associated with estrogen deficiency; however, it has been associated with an elevated risk of certain malignancies^9,10^ and has not consistently demonstrated beneficial effects on arterial stiffness^11^. Although antihypertensive medications are effective in managing high blood pressure, their long-term efficacy may be influenced by factors such as patient adherence, disease progression, and concomitant comorbidities, which can lead to suboptimal blood pressure control over time^12–14^. Given these limitations, non-pharmacological interventions, particularly exercise, should be considered a first-line strategy^15,16^. Exercise has been shown to effectively improve cardiovascular health in postmenopausal women^17^. Aquatic exercise, in particular, offers a low-impact, safe, and effective modality within a thermally neutral and buoyant environment, which can substantially reduce exercise limitations caused by joint pain, muscle weakness, and fear of falling in this population^18^. However, the cardiovascular benefits of exercise are not uniform across all individuals. Hagberg et al. (2000) reported that approximately 25% of individuals do not exhibit reductions in blood pressure following standardized exercise training^19^. Furthermore, some studies have observed no change^20^ or even increases^21^ in blood pressure after exercise interventions.

Emerging evidence suggests that interindividual variability in response to exercise may be partly attributed to genetic polymorphisms in the angiotensin-converting enzyme (ACE) gene. ACE, a key component of the renin-angiotensin-aldosterone system (RAAS), plays a critical role in blood pressure regulation and cardiovascular homeostasis^22–25^. A common insertion/deletion (I/D) polymorphism in intron 16 of the ACE gene results in the presence of either an insertion (I) or deletion (D) allele of a 287-bp DNA fragment^26^. Individuals carrying the D allele exhibit approximately twice the plasma ACE activity compared to those with the I allele^27^, and the D allele is associated with increased levels of angiotensin II (Ang II)—a potent vasoconstrictor—as well as higher blood pressure^26^. To date, there is a paucity of research examining the effects of aquatic exercise on blood pressure, lipid profiles, and arterial stiffness across different ACE genotypes in postmenopausal women. Most existing studies have focused on land-based exercise interventions, and findings regarding genotype-specific cardiovascular responses to exercise remain inconsistent. For instance, Montrezol et al. (2019) demonstrated that after 16 weeks of resistance training (three sessions per week), only older adults carrying the I allele exhibited significant reductions in SBP and DBP^25^. In contrast, Zhang et al. (2002) found that after 10 weeks of aerobic cycling (three times per week), individuals with the I allele showed significant reductions in SBP, DBP, and MAP at both week 4 and week 10, whereas those with the DD genotype exhibited a reduction in SBP only at week 4^28^. Jones et al. (2006) reported that a short-term aerobic exercise program (7–8 days) failed to improve blood pressure in hypertensive adults regardless of ACE genotype^29^.

To our knowledge, no study has yet investigated the impact of aquatic high-intensity interval training (HIIT) on the four major lipid outcomes and arterial stiffness across different ACE genotypes in postmenopausal women. Moreover, evidence regarding blood pressure responses in this context remains inconclusive. Therefore, the present study aims to examine the differential effects of a 12-week aquatic HIIT program on blood pressure, lipid profiles, and arterial stiffness among postmenopausal women stratified by ACE genotype.

## Methods

### Participants

Participants were recruited through multiple channels, including community announcements, WeChat groups, and referrals from existing participants, which constitutes a convenience sampling method. Inclusion criteria were as follows: natural menopause for at least one year; no current use of ACE inhibitors; and clearance based on the Physical Activity Readiness Questionnaire (PAR-Q)^30^. Exclusion criteria included severe musculoskeletal disorders, cardiovascular or cerebrovascular diseases, metabolic disorders, and cognitive impairment. Sixty eligible participants were initially enrolled. Ten participants voluntarily withdrew from the study, and three were lost to follow-up. The final sample consisted of 47 postmenopausal women, including 25 with the ACE II genotype, 14 with the ID genotype, and 8 with the DD genotype. All participants provided written informed consent prior to study participation. The study was approved by the Research Ethics Committee of Nanjing Normal University (Approval number: NNU202407009) and registered with the Chinese Clinical Trial Registry (Registration number: ChiCTR2400087544, July 30, 2024).

### Study design and procedure

A non-randomized comparative study design was employed. During the baseline assessment, all participants underwent screening with the PAR-Q, body composition measurement, and medical history interview. Individuals who met eligibility criteria provided buccal mucosal cell samples for ACE genotyping. In the second assessment phase, arterial stiffness was evaluated using baPWV, and fasting venous blood samples were collected for lipid analysis. All measurements of blood pressure, baPWV, and lipid profiles were performed by trained technicians who were blinded to the participants’ ACE genotypes during data collection and analysis.

Based on ACE genotype, participants were classified into two groups: the II genotype group (*n* = 25) and the ID/DD genotype group (*n* = 22). Due to the relatively low frequency of the DD genotype, individuals with ID and DD genotypes were combined into a single group to enhance statistical power and align with previous studies investigating genotype-dependent responses to exercise^25,28^. Both groups completed an identical 12-week aquatic HIIT program, conducted three times per week for 40 min per session. All outcome measures were reassessed within one week following the final training session. Throughout the intervention period, participants were instructed to maintain their usual dietary and lifestyle habits, and to refrain from initiating any additional structured exercise programs.

### Exercise intervention protocol

Aquatic HIIT was performed in a standing position with a water depth of 1 m. The water temperature was maintained at 28 ± 1℃, ambient room temperature at 29 ± 3℃, and relative humidity at 72 ± 6.5%. Each 40-minute session consisted of a 6-minute warm-up, 30 min of main exercise (three 10-minute interval blocks), and a 4-minute cool-down.

Each 10-minute interval block consisted of 2 min of aquatic resistance-based strength training, 6 min of jump training, and 2 min of dynamic recovery. The strength training component included 30 s of rapid concentric contractions of the latissimus dorsi followed by 30 s of rest, immediately followed by 30 s of rapid pectoralis major contraction and another 30 s of rest. The jump training phase comprised four consecutive 30-second exercises: countermovement jumps, lunge jumps, jumping jacks, and single-leg hops, performed in sequence. Each exercise was followed by a 60-second active recovery period to promote recovery and postural stability. The final 2 min of the block were designated as dynamic recovery, during which participants performed slow walking and controlled breathing to facilitate transition to the next interval or to the cool-down phase.

Exercise intensity was monitored using heart rate reserve (HRR) and the rating of perceived exertion (RPE) scale^31^. Week 1 served as an adaptation phase, during which exercise was performed at a comfortable, low-effort intensity to facilitate mastery of movement techniques and pacing. From weeks 2 to 12 (formal training phase), intensity was guided by dual monitoring of HRR and RPE: warm-up was conducted at RPE 10–13; each 30-second plyometric jump training interval was performed at 75% HRR ± 5 Bpm (RPE 14–17); dynamic recovery was maintained at 50% HRR ± 5 Bpm (RPE 12–13); and for resistance-based strength training, participants were instructed to perform contractions at maximal voluntary speed against water resistance, targeting an RPE of approximately 17–19. All participants attended an average of 33.4 ± 1.9 sessions out of the scheduled sessions (92.7% adherence). The exercise intervention was conducted under the supervision of four certified instructors, resulting in a participant-to-instructor ratio of approximately 47 : 4.

### Outcome measurements and data collection

#### ACE genotyping

Buccal mucosal cells were collected using sterile swabs rubbed bilaterally against the inner cheek epithelium for at least 20 strokes and immediately transferred into preservation tubes for storage until analysis. The ACE I/D polymorphism (289 bp Alu insertion/deletion) was genotyped using a fluorescence-based real-time quantitative PCR (qPCR) assay. Amplification was performed on a thermo fisher ABI 7500 Real-Time PCR system (Thermo fisher Scientific, Waltham, MA, USA), with a commercially available human ACE gene typing kit (fluorescence PCR method; Hangzhou Biobay biotechnology Co., Ltd., Hangzhou, China). Allele-specific probes targeting the insertion (I) and deletion (D) variants were designed based on the reference sequence of the ACE gene and labeled with distinct fluorescent dyes: FAM (6-carboxyfluorescein) for the I allele and VIC (6-carboxy-X-rhodamine) for the D allele. During PCR, a fluorescent signal was generated only upon perfect hybridization of the probe to its complementary allele and subsequent cleavage by the 5′-nuclease activity of Taq DNA polymerase. The cycle threshold (Ct) value—the number of cycles required for the fluorescence signal to exceed the background threshold—was recorded for each sample. Genotypes were assigned based on the Ct values obtained from the amplification curves in the FAM and VIC channels. The thermal cycling protocol is detailed in Table 1, and the genotype classification criteria are presented in Table 2.

**Table 1:** Real-Time fluorescent PCR amplification Protocol.

Step	Temperature	Time	Notes
1	95℃	5 min	Initial denaturation
2	95℃	15 s	40 amplification cycles
3	60℃	34 s	Annealing and fluorescence acquisition < sup > a

^a^Fluorescence signal collection occurred at 60 °C during each cycle.

**Table 2:** Genotyping criteria for the ACE I/D Polymorphism.

Result	FAM Channel (I allele)	VIC Channel (D allele)	ACE (I/D) Genotype
1	Ct ≤ 35	Ct > 35 or undetermined	Insertion homozygote (I/I)
2	Ct ≤ 35	Ct ≤ 35	Heterozygote (I/D)
3	Ct > 35 or undetermined	Ct ≤ 35	Deletion homozygote (D/D)

FAM, 6-carboxyfluorescein; VIC, 6-carboxy-X-rhodamine; Ct, cycle threshold; I, insertion; D, deletion.

#### Heart rate

Heart rate was continuously monitored using a waterproof heart rate sensor (Polar H10, Polar Electro, Kempele, Finland), secured horizontally around the lower thorax beneath the pectoral muscles. Data were wirelessly transmitted to a wrist-worn sport watch (Garmin forerunner 265, Garmin Ltd., Schaffhausen, Switzerland) for real-time feedback and session-based recording during each exercise session.

#### Body composition assessment

Body composition was assessed using a multi-frequency bioelectrical impedance analyzer (InBody770, inbody Co., Ltd., Seoul, South Korea). Participants were instructed to abstain from food intake and avoid vigorous physical activity for at least 1 h before measurement. Measurements were performed with participants standing barefoot on the device’s foot electrodes, hands gripping the hand electrodes, and arms slightly abducted from the torso. All metal objects (e.g., watches, jewelry) were removed, and participants wore light clothing to minimize interference with current conduction. To ensure optimal electrode contact, palms and soles were cleaned with an alcohol wipe before testing.

#### Blood pressure assessment

SBP and DBP were measured on the left upper arm at the brachial artery using the blood pressure automatic device (HEM-7121, OMRON Corporation, Kyoto, Japan). Participants were instructed to sit quietly in a comfortable chair with back support, with their feet flat on the floor and their arms supported at heart level. Before measurement, participants rested in a seated position for at least 5 min in a quiet, temperature-controlled environment. MAP was calculated using the standard formula: MAP = DBP + ⅓(SBP − DBP)^32^. Measurements were performed by trained personnel under standardized conditions to ensure reliability and consistency.

#### Lipid profile analysis

Fasting venous blood samples were collected to assess the lipid profile, including total cholesterol (TC), TG, HDL-C, and LDL-C. Participants were instructed to fast for at least 12 h prior to blood draw and to abstain from smoking and vigorous physical activity for 30 min before sampling. Blood collection was scheduled between 08:30 and 09:00 at a designated community health center. After a 10-minute seated rest, 3–5 mL of venous blood was drawn by trained community physicians. Samples were immediately stored at 2–8 °C for no more than 24 h before analysis. Lipid concentrations were measured using an Olympus AU2700 automated biochemical analyzer (Olympus optical Co., Ltd., Tokyo, Japan). Assays for HDL-C and LDL-C were performed using direct method reagent kits (Mindray Bio-Medical electronics Co., Ltd., Shenzhen, China). At the same time, TC and TG were analyzed with enzymatic oxidation method kits (Hangzhou Biobay biotechnology Co., Ltd., Hangzhou, China). The coefficient of variation for internal quality control was 3.0% for TC, 3.0% for TG, 2.5% for HDL-C, and 2.5% for LDL-C, reflecting high analytical precision. All measurements were conducted according to the manufacturer’s instructions using enzymatic colorimetric assays. Absorbance was read at wavelength-specific channels, and concentrations were automatically calculated by the instrument based on factory-calibrated standard curves.

#### Arterial stiffness assessment

BaPWV was measured using the automated vascular screening system BP-203RPE III (OMRON Corporation, Kyoto, Japan), a validated device for assessing arterial stiffness. Participants Lay supine in a quiet, temperature-controlled environment after at least 10 min of rest. Four pneumatic cuffs were placed on both upper and lower limbs: the upper-arm cuffs were positioned around the biceps region to detect brachial artery pressure waves, and the ankle cuffs were secured around the malleolar area to record posterior tibial artery pressure. Electrocardiographic electrodes were attached to the wrists (after skin cleansing with alcohol), and a microphone-type phonocardiographic sensor was placed at a point 2–3 cm left of the fourth intercostal space and gently pressed to optimize heart sound detection. BaPWV was computed as (*La* − *Lb*)/*ΔT*, where *La* and *Lb* are the distances from the aortic valve to the ankle and brachial artery, respectively, and *ΔT* is the pulse wave transit time between the two sites. All measurements were performed by trained technicians under standardized conditions.

### Statistical analysis

Data were processed and analyzed using IBM SPSS statistics version 22.0 (IBM Corp., Armonk, NY, USA). For continuous variables, normality was first assessed using the Kolmogorov-Smirnov test. Homogeneity of variances was evaluated with levene’s test prior to parametric testing. To account for baseline differences between groups, between-group comparisons of post-intervention values for all outcomes were conducted using analysis of covariance (ANCOVA), with the respective baseline value of the outcome as a covariate. For variables that Met the assumptions of normality and homogeneity of variances, within-group changes from baseline to post-intervention were assessed using paired t-tests (or Wilcoxon signed-rank tests for non-normally distributed data). Categorical variables, including the prevalence of hypertension and the rate of antihypertensive medication use, were analyzed using the chi-square (χ²) test. All statistical tests were two-tailed, and the level of significance was set at α = 0.05. Missing data were handled using an intention-to-treat (ITT) analysis approach. All available data from participants who completed at least one session were included in the analysis. A priori power analysis was conducted using G power software (Heinrich heine university Düsseldorf, Düsseldorf, Germany). Based on a medium effect size (d = 0.516), a significance level of α = 0.05, and a desired statistical power of 0.80, the calculated sample size required was 60 participants per group (total *n* = 120). The final sample of 47 participants is smaller than this requirement.

## Results

### Descriptive characteristics of participants

- There were no statistically significant differences between the II and ID/DD groups in age, age at menopause, height, body weight, body mass index (BMI), body fat percentage, skeletal muscle mass, prevalence of hypertension, or baseline use of antihypertensive medication (all *p* > 0.05). However, after the 12-week intervention, both groups showed significant increases in body weight, BMI, and body fat percentage compared with pre-intervention values (*p* < 0.05), although no significant between-group differences were observed in these changes (Table 3).
- Comparison of Blood Pressure and Arterial Stiffness Between II and ID/DD Genotype Groups After Exercise Training.

**Table 3:** Baseline and Post-Intervention descriptive characteristics of Participants.

Outcome Variable	Genotype Group	Mean ± SD (Pre-intervention)	Mean ± SD (Post-intervention)
Age (yrs)	II	59.0 ± 5.52	-
	ID/DD	57.4 ± 7.52	-
Age at menopause (yrs)	II	50.3 ± 3.64	-
	ID/DD	49.7 ± 4.41	-
Height (cm)	II	158.6 ± 4.29	-
	ID/DD	157.7 ± 5.11	-
Weight (kg)	II	61.4 ± 9.06	62.5 ± 9.11†
	ID/DD	60.2 ± 7.03	61.7 ± 7.93†
BMI (kg/m^2^)	II	24.4 ± 3.03	24.7 ± 3.29†
	ID/DD	24.2 ± 2.33	24.7 ± 2.85†
Body fat percentage (%)	II	32.2 ± 5.18	34.1 ± 4.67†
	ID/DD	31.8 ± 5.20	33.8 ± 5.24†
Skeletal muscle mass (kg)	II	22.6 ± 3.05	22.2 ± 2.67
	ID/DD	22.4 ± 2.65	22.0 ± 2.53
Hypertension (%)	II	37	-
	ID/DD	22.2	-
Antihypertensive medication use (%)	II	25.9	-
	ID/DD	18.5	-

II, insertion/insertion genotype; ID/DD, combined insertion/deletion and deletion/deletion genotypes; BMI: body mass index; †, significant within-group change from baseline (*p* < 0.05).

Following a 12-week aquatic HIIT program, there were no statistically significant differences in post-intervention SBP, DBP, or MAP between the II group and the ID/DD group after adjusting for baseline values (all *p* > 0.05). For arterial stiffness, although no significant between-group differences were observed in post-intervention baPWV on the right side (*p* = 0.058) or left side (*p* = 0.086), a trend toward greater baPWV values in the ID/DD group was noted after the intervention.

Within the II group, SBP, DBP, and MAP all significantly decreased from baseline to post-intervention (*p* = 0.023, 0.041, and 0.020, respectively), while baPWV (right) significantly increased (*p* = 0.019). In contrast, within the ID/DD group, both baPWV (right) and baPWV (left) significantly increased from baseline to post-intervention (*p* = 0.013 and 0.002, respectively) (Table 4).

**Table 4:** Comparison of blood pressure and arterial stiffness between II and ID/DD genotype groups after aquatic HIIT.

Outcome Variable	Genotype Group	Mean ± SD (Pre-intervention)	Mean ± SD (Post-intervention)	Adjusted Mean (95% CI)	*p*-value (ANCOVA)
SBP (mmHg)	II	130.7 ± 16.59	126.0 ± 15.30†	122.7 (118.8–126.6.8.6)	0.733
	ID/DD	121.4 ± 18.99	122.2 ± 17.22	125.8 (121.9–129.7.9.7)	
DBP (mmHg)	II	83.0 ± 9.88	79.4 ± 10.82†	77.5 (74.5–80.5)	0.543
	ID/DD	77.6 ± 9.71	79.7 ± 11.01	82.4 (79.1–85.6)	
MAP (mmHg)	II	98.9 ± 11.10	95.0 ± 11.39†	92.5 (89.4–95.6)	0.980
	ID/DD	92.2 ± 12.14	93.6 ± 12.16	96.5 (93.1–99.8)	
baPWV (cm/s) (Right)	II	1461.5 ± 275.16	1549.8 ± 368.96†	1512.6 (1443.4–1581.8.4.8)	0.058
	ID/DD	1395.0 ± 203.69	1494.4 ± 228.12†	1521.6 (1447.3–1596.0)	
baPWV (cm/s) (Left)	II	1479.1 ± 280.78	1555.6 ± 389.82‡	1509.1 (1437.0–1581.2.0.2)	0.086
	ID/DD	1395.7 ± 210.90	1500.0 ± 218.97†	1536.3 (1458.5–1614.2.5.2)	

The adjusted means were estimated from the ANCOVA model with baseline values as a covariate; The *p*-values are derived from the ANCOVA analysis for the genotype group effect; CI, confidence interval; HIIT, high-intensity interval training; II, insertion/insertion genotype; ID/DD, combined insertion/deletion and deletion/deletion genotypes; SBP, systolic blood pressure; DBP, diastolic blood pressure; MAP: mean arterial pressure; baPWV, brachial-ankle pulse wave velocity; †, significant within-group change from baseline (*p* < 0.05); ‡, trend toward a significant within-group change (*p* < 0.10).

### Comparison of lipid profile between II and ID/DD genotype groups after exercise training

The adjusted mean TG level after the intervention was significantly lower in the ID/DD group than in the II group (*p* = 0.000). A trend toward higher HDL-C levels was observed in the ID/DD group compared to the II group after the intervention (*p* = 0.053).

Within-group analyses revealed that HDL-C significantly increased and LDL-C significantly decreased from baseline to post-intervention in both groups (*p* < 0.05), while no significant changes were observed in TC or TG levels within either group (Table 5).

**Table 5:** Comparison of lipid profile between II and ID/DD genotype groups after aquatic HIIT.

Outcome variable	Genotype group	Mean ± SD (Pre-intervention)	Mean ± SD (Post-intervention)	Adjusted Mean (95% CI)	*p*-value (ANCOVA)
TC (mmol/L)	II	5.36 ± 0.78	5.31 ± 0.82	5.37 (5.11–5.63)	0.380
	ID/DD	5.52 ± 0.94	5.31 ± 0.90	5.26 (4.99–5.53)	
HDL-C (mmol/L)	II	1.53 ± 0.28	1.73 ± 0.32†	1.75 (1.67–1.83)	0.053
	ID/DD	1.57 ± 0.39	1.79 ± 0.33†	1.78 (1.69–1.87)	
LDL-C (mmol/L)	II	3.56 ± 0.64	3.11 ± 0.61†	3.15 (2.92–3.38)	0.311
	ID/DD	3.71 ± 0.91	3.18 ± 0.70†	3.15 (2.91–3.40)	
TG (mmol/L)	II	1.44 ± 0.94	1.54 ± 1.50	1.66 (1.43–1.88)	0.000
	ID/DD	1.61 ± 0.80	1.33 ± 0.45‡	1.31 (1.07–1.55)	

The adjusted means were estimated from the ANCOVA model with baseline values as a covariate; The p-values are derived from the ANCOVA analysis for the genotype group effect; CI, confidence interval; HIIT, high-intensity interval training; II, insertion/insertion genotype; ID/DD, combined insertion/deletion and deletion/deletion genotypes; TC, total cholesterol; LDL-C, low-density lipoprotein cholesterol; HDL-C, high-density lipoprotein cholesterol; TG, triglyceride; †, significant within-group change from baseline (*p* < 0.05); ‡, trend toward a significant within-group change (*p* < 0.10).

## Discussion

The present study investigated the differential effects of a 12-week aquatic HIIT program on cardiovascular physiological and biochemical outcomes in postmenopausal women with the ACE II genotype compared to those with the ID/DD genotype. After adjusting for baseline values using ANCOVA, no statistically significant differences were observed in post-intervention SBP, DBP, MAP, or baPWV between the two groups. However, within-group analyses revealed that SBP, DBP, and MAP all significantly decreased from baseline to post-intervention in the II group, while baPWV (right) significantly increased. In contrast, both baPWV (right) and (left) significantly increased from baseline to post-intervention in the ID/DD group.

### Genotype-specific responses in blood pressure regulation to exercise training

Hypertension is a major risk factor for accelerated vascular remodeling and arterial stiffening^33,34^. In the present study, although ANCOVA-adjusted between-group comparisons revealed no statistically significant differences in SBP, DBP, or MAP between ACE genotype groups, within-group analyses demonstrated significant reductions in all three indices among II genotype carriers following the 12-week aquatic HIIT intervention. In contrast, ID/DD carriers exhibited minimal or non-significant changes—consistent with the well-documented phenomenon of “non-response” to exercise, which affects approximately 25% of individuals^19^. Our findings align with prior evidence indicating that the hypotensive benefits of exercise are most pronounced in I allele carriers. For instance, Montrezol et al. (2019) reported significant reductions in 24-hour and daytime SBP/DBP after 16 weeks of resistance training in older adults carrying the I allele^25^, while Zhang et al. (2002) observed sustained declines in SBP, DBP, and MAP over 10 weeks of aerobic cycling exclusively in I allele carriers, with only transient effects in DD individuals^28^.

These genotype-dependent responses likely stem from the functional impact of the ACE I/D polymorphism on the RAAS^24,35^. I allele carriers exhibit lower ACE activity, resulting in reduced conversion of angiotensin I to the potent vasoconstrictor Ang II and consequently lower circulating Ang II levels^27^, which may contribute to more favorable blood pressure responses following long-term exercise. Additionally, differences may involve nitric oxide (NO) synthesis and downregulation of sympathetic nervous activity. Due to lower Ang II levels, II genotype carriers may exhibit greater sensitivity to endothelium-dependent vasodilation. Exercise-induced increases in NO release may therefore be more pronounced, leading to enhanced vasodilation, reduced vascular resistance, and improved blood pressure regulation^36^. Previous research indicates that acute exercise may transiently stimulate renin release, which converts hepatic angiotensinogen to Ang I. Ang I is then converted to the potent vasoconstrictor Ang II by ACE. However, individuals with the I allele have lower ACE activity, limiting Ang II generation and enhancing overall suppression of the RAAS, thereby amplifying the hypotensive effects of exercise^36,37^. Regular exercise can also reduce sympathetic nervous system activity^38^, decrease β1-receptor activation on juxtaglomerular cells, suppress renin secretion^39,40^, and enhance baroreflex sensitivity^41^, thereby improving blood pressure regulation. These beneficial adaptations may be more evident in individuals with the II genotype. Notably, blood pressure indicators in the II group were significantly lower than those before intervention, while the II/DD group remained unchanged. This difference may reflect a clinically relevant trend, potentially limited by our sample size. The blunted response in D allele carriers is plausibly attributable to elevated ACE activity and heightened Ang II production, which may counteract exercise-induced vasodilation and sympathetic modulation^36,37^.

Collectively, these results underscore the potential of ACE genotype as a modifier of exercise responsiveness in blood pressure regulation and support the emerging paradigm of genotype-informed exercise prescription for hypertension management in aging populations.

### Genotype-specific changes in arterial stiffness

BaPWV is a well-established non-invasive index for assessing arterial stiffness^42^. In the present study, both ACE II and ID/DD groups exhibited increases in baPWV following the 12-week aquatic HIIT intervention. Notably, while within-group analyses revealed significant elevations in right baPWV among II carriers and significant increases in both right and left baPWV among ID/DD carriers, ANCOVA-adjusted comparisons indicated a trend toward higher post-intervention baPWV values in the ID/DD group relative to the II group. These findings are supported by Fujie et al. (2025), who reported that 12 weeks of high-intensity resistance training in postmenopausal women led to a significant increase in carotid-femoral pulse wave velocity—a key marker of arterial stiffness—and demonstrated a positive correlation between this change and elevated Ang II levels^43^. These results suggest that the ACE gene polymorphism may modulate differential vascular adaptations to exercise through pathways involving Ang II. Notably, Ang II has been shown to promote premature senescence of vascular smooth muscle cells, which may contribute to the absence of improvement—or even progression—of arterial stiffness^44,45^. This perspective is further supported by Salviano de Faria et al. (2022), who reported a significant increase in Ang II levels after 10 weeks of lower-limb resistance training^6^. The present findings extend these results by demonstrating that, despite differences in modality—aquatic HIIT versus land-based high-intensity resistance training—both forms of exercise involve rapid, forceful muscle contractions against external resistance, which may similarly activate the RAAS. Specifically, the aquatic HIIT protocol included rapid lower-limb jumps and upper-limb resistance exercises performed in water, both of which require participants to overcome body weight, hydrostatic pressure, and drag forces. These mechanical challenges may contribute to activation of the RAAS, leading to increased Ang II levels. Importantly, while both genotype groups exhibited increases in baPWV following intervention, the magnitude and statistical significance of this response differed: ID/DD carriers showed significant elevations in both right and left baPWV, whereas II carriers exhibited a significant increase only in right baPWV and a trend toward increase in left baPWV. Moreover, ANCOVA-adjusted post-intervention values tended to be higher in the ID/DD group compared to the II group (right baPWV: *p* = 0.058; left: *p* = 0.086). This suggests that although exercise-induced RAAS activation may elevate arterial stiffness in all individuals, the D allele confers heightened susceptibility to this adverse vascular adaptation—likely due to genetically determined higher baseline ACE activity and Ang II production^27,36^. In contrast, the I allele’s association with lower ACE activity may partially buffer against exercise-induced Ang II surges, thereby attenuating the degree of arterial stiffening—even if not fully preventing it^46^. A previous systematic review and meta-analysis confirmed that high-intensity resistance training is associated with increased arterial stiffness, whereas moderate-intensity training does not exhibit this effect^47^. In the present study, both the 30-second bouts of rapid jumping and the 30-second upper-limb movements against water resistance were performed at high intensity. Therefore, it is plausible that the resistive components inherent in aquatic HIIT—particularly those involving explosive, eccentric, or isometric muscle actions—may contribute to the observed elevation in baPWV. However, the genotype-specific modulation of this response underscores the importance of individualized exercise prescription: for individuals carrying the D allele, strategies to mitigate RAAS overactivation (e.g., concurrent aerobic training, pharmacological support, or intensity modulation) may be warranted to preserve vascular health.

### General improvements in lipid profile and genotype-modulated responses

LDL-C, HDL-C, TC, and TG are key indicators for assessing cardiovascular health and metabolic status, providing critical information for the diagnosis, treatment, and prevention of atherosclerotic diseases, coronary heart disease, and stroke^48^. LDL-C is recognized as a major modifiable risk factor for cardiovascular disease and has been causally linked to atherosclerotic cardiovascular disease^49^. Following the 12-week aquatic high-intensity interval-training programme, LDL-C level declined significantly and HDL-C level increased significantly from baseline in postmenopausal women of both genotypes groups. These findings are consistent with previous studies. Kim et al. (2021) reported that postmenopausal women who completed 12 weeks of moderate-to-high-intensity aquatic aerobic exercise (three sessions per week, RPE 12–15) experienced a 23.08% decrease in LDL-C and a 9.84% increase in HDL-C^50^. Similarly, Woo-Cheol et al. (2016) observed a 9.88% reduction in LDL-C and a 27.4% increase in HDL-C following 12 weeks of moderate-intensity water-based aerobic training in older women with hypertension^51^. Furthermore, systematic meta-analyses support these results, confirming that aquatic exercise exerts favorable effects on LDL-C and HDL-C levels in postmenopausal women^52,53^. The beneficial changes in LDL-C and HDL-C may be mediated by multiple mechanisms, including enhanced reverse cholesterol transport, increased lipoprotein lipase activity, improved insulin sensitivity, and upregulation of low-density lipoprotein receptor mRNA expression in hepatocytes. Together, these adaptations accelerate the hepatic clearance and metabolism of LDL-C^54^. Interestingly, the improvement in HDL-C observed in the present study contrasts with our previous findings. Our systematic review and meta-analysis indicated that no significant improvement in HDL-C was associated with aquatic exercise in postmenopausal women^55^. However, prior evidence suggests that exercise-induced increases in HDL-C may require a minimum intensity threshold — specifically, at least 75% of maximal heart rate^56^. Given that the current study employed a HIIT protocol, it is plausible that the higher exercise intensity contributed to the observed HDL-C elevation. Moreover, the unique hydrodynamic environment of aquatic exercise may enhance venous return and cardiac output, potentially amplifying the metabolic stimulus for HDL-C synthesis. Importantly, while the improvement in LDL-C and HDL-C did not differ significantly between genotype groups—suggesting that the beneficial effects of aquatic HIIT on these lipids are largely independent of ACE I/D polymorphism—post-intervention lipid profiles revealed genotype-dependent patterns: adjusted mean TG levels were significantly lower in the ID/DD group compared to the II group (*p* = 0.000), and HDL-C levels showed a trend toward being higher in the ID/DD group (*p* = 0.053). This indicates that while exercise-induced improvements in LDL-C and HDL-C may be universally achievable across genotypes, the final lipid profile—particularly TG and HDL-C levels—may be modulated by genetic background. The distinct metabolic response observed in ID/DD carriers suggests a genotype-specific adaptation mechanism. While D allele carriers typically exhibit elevated ACE activity and Ang II level—which are often linked to insulin resistance and dyslipidemia—acute or chronic exercise may differentially modulate these pathways^57^. Specifically, physical activity might enhance hepatic clearance of TG or activate lipolytic enzymes in this subgroup. Furthermore, the ID/DD genotype may be associated with greater adipose tissue responsiveness to catecholamine-driven lipolysis during high-intensity exercise, potentially facilitating enhanced TG mobilization^58^. Future research is needed to elucidate whether these beneficial responses are mediated by RAAS modulation, sympathetic nervous system activation, or other metabolic regulators.

### Limitations and implications

Several limitations should be acknowledged in the present study. First, the non-randomized design may have introduced confounding factors that could obscure genotype-specific effects. Second, the relatively small sample size may have limited statistical power to detect subtle, genotype-dependent differences. Third, the high prevalence of hypertension among participants and the lack of systematic dietary records may affect the generalizability of the findings, limiting their applicability to broader populations. Fourth, the absence of molecular-level measurements—such as Ang II, bradykinin, and other components of the RAAS—precludes definitive conclusions regarding RAAS activation and its specific contribution to gene-exercise interactions. Fifth, although we adjusted for baseline values using ANCOVA, the absence of a true control group limits our ability to draw causal inferences about the effects of the intervention. Sixth, the use of convenience sampling may limit the representativeness of the cohort.

The findings of this study have important implications for cardiovascular health management in postmenopausal women. First, aquatic HIIT is a low-impact and relatively safe exercise modality that aligns well with the age- and physiology-related characteristics of postmenopausal women, making it a suitable physical activity option for this population. Second, while both genotype groups experienced significant improvements in HDL-C and LDL-C, ID/DD carriers demonstrated a more favorable triglyceride response, with significantly lower post-intervention TG levels compared to II carriers—suggesting that individuals with the D allele may derive unique metabolic benefits from this training modality. Third, although the II genotype group exhibited significant within-group reductions in blood pressure, both groups showed increases in arterial stiffness, with ID/DD carriers displaying greater elevations in baPWV on both sides. This indicates that aquatic HIIT may entail potential vascular trade-offs: favorable lipid and blood pressure adaptations coexisting with possible adverse effects on arterial compliance. Therefore, routine monitoring of arterial stiffness during high-intensity aquatic training programs is warranted, particularly in postmenopausal women carrying the D allele, to balance cardiometabolic benefits against vascular risks.

## Conclusions

The aquatic HIIT program was associated with favorable improvements in blood lipids in postmenopausal women, with both genotype groups showing beneficial changes. However, no significant ACE genotype-specific effects were observed on blood pressure or arterial stiffness after adjusting for baseline values. While the II group exhibited favorable reductions in blood pressure, the ID/DD group demonstrated a significant reduction in TG and a trend toward higher HDL-C levels, suggesting potential metabolic benefits. Notably, both groups showed increased arterial stiffness following the intervention, indicating a potential vascular risk. These findings suggest the need for monitoring arterial stiffness during aquatic HIIT.

## Data Availability

The data that support the findings of this study are available from the corresponding author on reasonable request.

## Abbreviations

SBP: Systolic blood pressure
DBP: Diastolic blood pressure
MAP: Mean arterial pressure
LDL-C: Low-density lipoprotein cholesterol
HDL-C: High-density lipoprotein cholesterol
TC: Total cholesterol
TG: Triglycerides
baPWV: Brachial-ankle pulse wave velocity
HIIT: High-intensity interval training
HRR: Heart rate reserve
RPE: Rating of perceived exertion
PAR-Q: Physical activity readiness questionnaire
Mean ± SD: Mean ± standard deviation
ACE: Angiotensin-converting enzyme
II: Insertion/insertion genotype
ID/DD: Insertion/deletion and deletion/deletion genotypes
RAAS: Renin-angiotensin-aldosterone system
Ang II: Angiotensin II
NO: Nitric oxide
Ct: Cycle threshold
